# HBP‐A Attenuates Knee Osteoarthritis Progression via MLK3/P38/HDAC4 Axis‐Mediated Dual Protection of Articular Cartilage and Quadriceps

**DOI:** 10.1111/jcmm.70577

**Published:** 2025-05-03

**Authors:** Zhengming Wang, Junyan Huang, Yuanyuan Feng, Zhengyan Li, Haiya Ge, Rui Wang, Yong Gu, Yizhe Xiong, Bo Chen, Mingcai Zhang, Xiang Wang, Ying Shi, Zhibi Shen, Hongsheng Zhan, Guoqing Du

**Affiliations:** ^1^ Shi's Center of Orthopedics and Traumatology Shuguang Hospital Affiliated to Shanghai University of Traditional Chinese Medicine Shanghai China; ^2^ Institute of Traumatology & Orthopedics Shanghai Academy of Traditional Chinese Medicine Shanghai China; ^3^ Department of Medical Oncology Shuguang Hospital Affiliated to Shanghai University of Traditional Chinese Medicine Shanghai China; ^4^ Department of Orthopedic Surgery Shanghai Guanghua Hospital of Integrated Traditional Chinese and Western Medicine Shanghai China; ^5^ Translational Medical Innovation Center Zhangjiagang TCM Hospital Affiliated to Nanjing University of Chinese Medicine Zhangjiagang Jiangsu China

**Keywords:** cartilage, Hyriopsis Bioactive Polysaccharide‐Anodonta, MLK3/P38/HDAC4 axis, osteoarthritis, quadriceps

## Abstract

Knee osteoarthritis (KOA), a degenerative joint disease driven by biomechanical instability, involves cartilage degradation, muscle dysfunction, and MLK3/P38 MAPK pathway activation. Histone deacetylase 4 (HDAC4), a regulator of chondrocyte and muscle homeostasis, interacts with this pathway during disease progression. While Hyriopsis Bioactive Polysaccharide‐Anodonta (HBP‐A) exhibits P38 MAPK inhibitory properties in vitro, its in vivo therapeutic effects on musculoskeletal tissues remain uncharacterised. A destabilisation of the medial meniscus (DMM) mouse model was established to investigate HBP‐A's therapeutic potential. Animals were randomly divided into sham‐operated, DMM‐induced, and HBP‐A‐treated groups. Following surgical induction, HBP‐A (0.26 g/kg) was administered daily via oral gavage for 4 weeks. Comprehensive assessments included behavioural tests for pain sensitivity, micro‐CT scanning, histological evaluation, and transmission electron microscope. Molecular mechanisms were investigated via immunohistochemical or immunofluorescence staining of MLK3, P38 MAPK, Caspase‐3, and HDAC4, complemented by RT‐qPCR analysis of myokine expression. HBP‐A treatment significantly alleviated pain sensitivity compared to the DMM group. Structural evaluations revealed preserved subchondral bone integrity and attenuated cartilage degeneration, with histological scoring confirming reduced pathological changes. Quadriceps exhibited mitigated atrophy and restored ultrastructural organisation. Molecular profiling demonstrated suppressed MLK3/P38 MAPK pathway activation, diminished apoptotic activity, and elevated HDAC4 expression in both cartilage and quadriceps. HBP‐A additionally normalised dysregulated expression of muscle‐derived osteogenic factors linked to bone‐cartilage crosstalk. These findings establish HBP‐A as a dual‐target therapeutic agent for KOA, concurrently mitigating cartilage and muscle degeneration through MLK3/P38 MAPK/HDAC4 axis modulation.

**Abbreviations**
BV/TV: bone volume/tissue volume
COL‐2: collagen type II
DMM: destabilisationdestabilization of the medial meniscus
FGF‐2: fibroblast growth factor 2
HBP‐A: Hyriopsis Bioactive Polysaccharide Anodonta
HDAC4: histone deacetylase 4
H&E: haematoxylinhematoxylin‐–eosin
HYC: Huaizhen Yanggan Capsule
IF: Immunofluorescence
IGF‐1: insulin‐like growth factor 1
IHC: Immumohistochemical
KOA: knee osteoarthritis
micro‐CT: Micro‐computed tomography
MLK3: mixed‐lineage kinase 3
MMP‐13: matrix metalloproteinase 13
MMP‐3: matrix metalloproteinase‐3
MMTL: medial meniscotibial ligament
MSTN: myostatin
OA: Osteoarthritis
P38 MAPK: P38 mitogen‐activated protein kinase
PWL: paw withdrawal latency
PWT: paw withdrawal threshold
RT‐qPCR: Reverse transcription‐quantitative polymerase chain reaction
S.O.: Safranin O‐Fast Green
Tb.N: trabecular number
Tb.Sp: trabecular separation
Tb.Th: trabecular thickness
TCM: Traditional Chinese Medicine
TEM: Transmission Electron Microscopy

## 1 Introduction

Osteoarthritis (OA) represents a major global health burden, affecting approximately 500 million individuals worldwide and ranking as the most prevalent degenerative joint disorder [1]. The disease manifests through progressive articular cartilage degradation and chronic pain, with its incidence escalating due to population ageing and rising obesity rates—both established risk factors that amplify disease severity [2]. Among OA subtypes, knee osteoarthritis (KOA) demonstrates particular clinical significance, accounting for a substantial proportion of OA‐related disability and substantially impacting patients' quality of life [3]. The current understanding of KOA pathogenesis emphasises multifactorial interactions between systemic predispositions (e.g., genetic susceptibility, metabolic dysregulation) and local biomechanical stressors that disrupt joint homeostasis [4, 5]. Emerging evidence highlights the pivotal role of biomechanical instability in KOA development and progression [6, 7, 8, 9]. Specifically, the interplay among cartilage degradation, altered bone remodelling, and abnormalities in surrounding soft tissues disrupts joint homeostasis, perpetuating a feedback loop that accelerates disease progression [10].

Therapeutic strategies increasingly focus on dual targeting of articular cartilage preservation and quadriceps muscle rehabilitation [11]. Cartilage degeneration, a central pathological feature of KOA, results from disrupted equilibrium between anabolic and catabolic processes mediated through cytokine networks and enzymatic cascades [12]. Concurrently, quadriceps dysfunction is closely linked to KOA progression, as it impairs the joint's ability to manage mechanical loads, creating a self‐perpetuating cycle of mechanical stress and structural deterioration [13]. Recent discoveries [14, 15, 16] regarding skeletal muscle's secretory function have revealed novel therapeutic dimensions, with myokines including Irisin, insulin‐like growth factor 1 (IGF‐1), fibroblast growth factor 2 (FGF‐2), and myostatin (MSTN) demonstrating regulatory effects on subchondral bone homeostasis—a critical component in KOA pathogenesis [17]. This dual biomechanical‐biochemical role positions the quadriceps muscle as a pivotal therapeutic target warranting comprehensive investigation.

The P38 mitogen‐activated protein kinase (P38 MAPK) signalling pathway has emerged as a crucial mediator of cellular stress responses in mechanically loaded joint tissues, regulating key processes such as inflammation, apoptosis, and chondrocyte differentiation [18, 19]. Experimental evidence [20, 21] has demonstrated the involvement of the P38 MAPK pathway in the pathogenesis of KOA, showing that inhibition of P38 MAPK can reduce inflammatory responses and cartilage destruction, thus highlighting its potential as a therapeutic target. Moreover, mixed‐lineage kinase 3 (MLK3), a crucial upstream kinase in the P38 MAPK signalling pathway, functions as a MAP3K that connects extracellular mechanical stimuli to intracellular stress responses [22]. It has been reported that MLK3 played an essential role in mediating bone formation and apoptosis [23, 24]. Furthermore, histone deacetylase 4 (HDAC4), a class IIa histone deacetylase predominantly expressed in musculoskeletal tissues, has been implicated in OA pathogenesis through its interactions with the P38 MAPK cascade [25]. Mechanistic studies [26, 27, 28] demonstrate that the progressive depletion of HDAC4 observed in osteoarthritic joints directly correlates with pathological upregulation of the Runx2 and MMP13 (matrix metalloproteinase 13), key mediators of cartilage degeneration, establishing a critical link between HDAC4 dysregulation and OA progression. Our preliminary study [29] using primary meniscus fibrochondrocytes further substantiates HDAC4's chondroprotective potential, while its established role in skeletal muscle development suggests broader regulatory functions in KOA pathophysiology [30].

Traditional Chinese Medicine (TCM), with its holistic approach and multi‐target therapeutic strategies, offers unique advantages in the treatment of KOA [31]. Therapies such as compound medicines, acupuncture, and massage have demonstrated significant efficacy in reducing pain, improving joint function, and slowing disease progression [32, 33]. As a result, TCM has become an integral component of KOA management, attracting increasing attention in clinical practice and research. Considering the characteristics of KOA, our research team developed a proprietary compound formulation, Huaizhen Yanggan Capsule (HYC), consisting exclusively of mussel meat. This formulation has been used as an exclusive prescription for over 20 years in clinical practice at our hospital. Through meticulous extraction processes, we identified Hyriopsis Bioactive Polysaccharide‐Anodonta (HBP‐A) as the active component and secured a Chinese national patent (ZL200610028598.0). HYC has demonstrated efficacy in reducing joint inflammation, protecting cartilage, and improving clinical symptoms in KOA patients [34]. Our previous studies [29, 35] revealed that HBP‐A delays meniscus hypertrophy and mineralisation, increases collagen type II (COL‐2) expression, and reduces matrix metalloproteinase‐3 (MMP‐3) and a disintegrin and metalloproteinase with thrombospondin motifs‐5 (ADAMTS‐5) levels in chondrocytes. These findings suggest its protective effects against cartilage degradation, a central feature of KOA pathophysiology. Despite these promising results, the specific effects of HBP‐A on cartilage and quadriceps degeneration, as well as the precise mechanisms underlying its actions, remain poorly characterised in vivo.

This study investigates the hypothesis that HBP‐A attenuates KOA progression through dual modulation of cartilage and quadriceps degeneration via the MLK3/P38/HDAC4 axis in a mechanically induced murine model. By elucidating these mechanisms, we aim to advance understanding of KOA pathophysiology and provide translational insights for developing novel therapeutic strategies.

## 2 Materials and Methods

### 2.1 Drugs

HBP‐A was provided by Shuguang Hospital, affiliated with Shanghai University of Traditional Chinese Medicine.

### 2.2 Mice and Model

In this study, 10‐week‐old male C57BL/6J mice were purchased from Shanghai Sippr‐BK Laboratory Animal Co Ltd. (Shanghai, China). Mice were group‐housed (six per cage) under a 12‐h light–dark cycle with *ad libitum* access to food and water. Based on a previous study [36], destabilisation of the medial meniscus (DMM) surgery was employed to induce KOA due to its reproducibility and ease of implementation. Eighteen mice were randomly assigned to three groups: the Sham group, DMM group, and HBP‐A group (*n* = 6 per group). Mice were anaesthetised intraperitoneally with pentobarbital sodium (0.5 mg/10 g body weight). A 0.5 cm incision was made from the distal patella to the proximal tibial plateau, followed by capsulotomy. The medial meniscotibial ligament (MMTL) of the right knee joint was completely dissected to induce medial meniscal instability. Postoperatively, mice were randomly divided into the HBP‐A group and the DMM group. In the Sham group, mice underwent capsulotomy without MMTL dissection. The HBP‐A group received HBP‐A dissolved in 0.9% normal saline (0.26 g/kg) by gavage, while the Sham and DMM groups received vehicle treatment (0.9% normal saline) once daily for 4 weeks, based on clinical dose conversions. Following the intervention, behavioural tests were conducted, after which mice were euthanised using an overdose of pentobarbital sodium. Knee joints and quadriceps were harvested for further analysis. All animal procedures complied with the Experimental Animal Ethics Committee of Shanghai University of Traditional Chinese Medicine.

### 2.3 Thermal Plantar Test

Paw withdrawal latency (PWL) was assessed using a thermal plantar test (Ugo Basile, Italy), as previously described [37]. Mice were individually placed in transparent‐bottom boxes in a quiet room for 30 min to minimise exploratory behaviour. An infrared heat stimulus (intensity = 60%) was applied to the mid‐plantar surface of the right hind paw, and PWL was automatically recorded. A cut‐off time of 20 s was set to prevent potential burn injuries. Hind paws were alternately tested with a 5‐min interval between trials. Five measurements per paw were averaged to determine the final result. The investigator was blinded to group assignments.

### 2.4 Von Frey Test

The 50% paw withdrawal threshold (PWT) was measured using Von Frey hairs (Touch Test, USA) and the up‐and‐down method, as previously described [38]. Mice were habituated for 30 min in individual boxes with metal mesh bottoms to reduce exploratory behaviour. Filaments with varying forces (starting at 0.16 g) were applied perpendicularly to the mid‐plantar surface of the right hind paw. A positive response (withdrawal, licking, or jumping) was recorded as ‘X’, and a weaker filament was applied; a negative response was recorded as ‘O’, followed by a stiffer filament. A sequence of six X and O responses was obtained to calculate the final PWT using Chaplan's formula [39]. The force range was set between 0.02 g and 2.0 g. The investigator remained blinded to group identity.

### 2.5 Micro‐Computed Tomography (Micro‐CT) Analyses

Knee joint samples were fixed in 4% paraformaldehyde (BL539A, Biosharp, Hefei, China) for 72 h prior to micro‐CT scanning. Scans were performed using a micro‐CT scanner (NEMO, Pingseng, Suzhou, China) with the following parameters: tube voltage = 60 kV, frame rate = 20/s, tube current = 120 μA, distance source‐detector (DSD) = 395 mm, and a transverse field of view = 15 mm. The scanning software (Cruiser, Pingseng) processed the images to assess bone volume/tissue volume (BV/TV), trabecular thickness (Tb.Th), trabecular separation (Tb.Sp), and trabecular number (Tb.N). The subchondral bone of the tibial plateau was selected as the region of interest. Two‐dimensional greyscale image slices were reconstructed into three‐dimensional tomograms using commercial software (Recon, Pingseng).

### 2.6 Histology

For histological analysis, knee joint samples were fixed in 4% paraformaldehyde for 72 h and decalcified in 14% ethylenediaminetetraacetic acid (EDTA) solution (pH 7.2) for 14 days. Skeletal muscle samples were freshly isolated and fixed in muscle fixation fluid (Servicebio, Wuhan, China) for 24 h. Subsequently, specimens were dehydrated using a graded ethanol series and embedded in paraffin. Paraffin blocks were sectioned sagittally and transversely into 4 μm‐thick slices for staining.

Following deparaffinisation and rehydration, sections were stained with haematoxylin–eosin (H&E) and Safranin O‐Fast Green (S.O.) to assess morphological features and proteoglycan expression in knee samples. Histopathological assessment was systematically performed according to the standardised Osteoarthritis Research Society International (OARSI) grading protocol [40]. Two blinded evaluators independently scored cartilage degeneration using the validated OARSI scale (Grades 0–6), with strict adherence to double‐blind principles to eliminate observational bias. The scoring system categorises pathological progression as follows: Grade 0 denotes normal cartilage architecture; Grades 1–2 involve superficial zone irregularities including surface fibrillation and early fissures; Grades 3–4 demonstrate progressive matrix loss with mid‐zone fissuring and partial‐thickness erosion; and Grades 5–6 represent advanced degeneration characterised by full‐thickness ulceration and subchondral bone exposure. Each grade incrementally reflects the depth of cartilage damage and associated cellular abnormalities. For quadriceps samples, H&E and Masson's trichrome staining were performed to observe morphology and assess collagen fibre distribution. The muscle fibre diameter and collagen fibre area were quantified using ImageJ software (NIH).

### 2.7 Ultrastructural Analysis (Transmission Electron Microscopy, TEM)

A segment of quadriceps muscle tissue was prepared for TEM analysis (Tecnai G2, FEI, USA) as previously described. Briefly, 1 mm^3^ tissue sections were fixed in 2% glutaraldehyde, followed by postfixation in 1% osmium tetroxide. After rinsing twice in buffer, samples were dehydrated through a graded ethanol series and embedded in epoxy resin. Ultra‐thin sections (~60 nm) were cut using a Leica Ultracut UCT ultramicrotome and stained with uranyl acetate for ultrastructural observation.

### 2.8 Immunohistochemical (IHC) and Immunofluorescence (IF) Staining

For IHC, paraffin‐embedded sections were deparaffinised in xylene and rehydrated in an ethanol gradient. Antigen retrieval was performed using sodium citrate at 60°C for 4 h. Samples were treated with an endogenous peroxidase blocker (Beyotime, Shanghai) at room temperature for 10 min, followed by incubation with 0.3% Triton X‐100 for 15 min. Sections were then incubated with primary antibodies at 4°C overnight, followed by treatment with corresponding secondary antibodies for 30 min the next day. Positive staining was visualised using diaminobenzidine (DAB) solution (Beyotime, Shanghai), and CAT haematoxylin was used for counterstaining.

For IF, sections were incubated with fluorescent secondary antibodies for 40 min at room temperature in the dark, and 4',6‐diamidino‐2‐phenylindole (DAPI) was used for nuclear staining. The primary antibodies used in this study included collagen type II (COL‐2) (1:200, Abcam, UK), MMP‐13 (1:200, Abcam, UK), Caspase‐3 (1:200, Proteintech, China), MLK 3 (1:100, Proteintech, China), phosphorylated P38 MAPK (P‐P38 MAPK) (1:100, Proteintech, China), and HDAC4 (1:200, GeneTex, USA). Quantitative analyses were performed using ImageJ software.

### 2.9 Reverse Transcription‐Quantitative Polymerase Chain Reaction (RT‐qPCR)

Muscle‐bone crosstalk plays a crucial role in maintaining joint homeostasis, while its dysregulation contributes to KOA progression. To investigate the effects of HYC on bone factors secreted by the quadriceps, RT‐qPCR was performed to quantify the expression of key bone factors, including Irisin, fibroblast growth factor 2 (FGF‐2), insulin‐like growth factor 1 (IGF‐1), and myostatin (MSTN). Total RNA was extracted from quadriceps tissues using TRIzol reagent (Sigma, USA). Reverse transcription was conducted using a cDNA Synthesis Kit (Takara, Dalian, China), and RT‐qPCR analysis was performed using SYBR Premix Ex Taq II (Takara, Dalian, China). β‐actin was used as the reference gene for normalisation. The primer sequences for the target genes are provided in Table S1.

### 2.10 Statistical Analysis

Data are presented as mean (M) ± standard deviation (SD). All statistical analyses were performed using SPSS version 20.0. One‐way analysis of variance (ANOVA) was used for comparisons involving more than two groups, followed by Dunnett's *t*‐test for pairwise comparisons. A *p*‐value < 0.05 was considered statistically significant.

## 3 Results

### 3.1 HBP‐A Ameliorated Pain and KOA Development in the DMM‐Induced Mice Model

**FIGURE 1:**
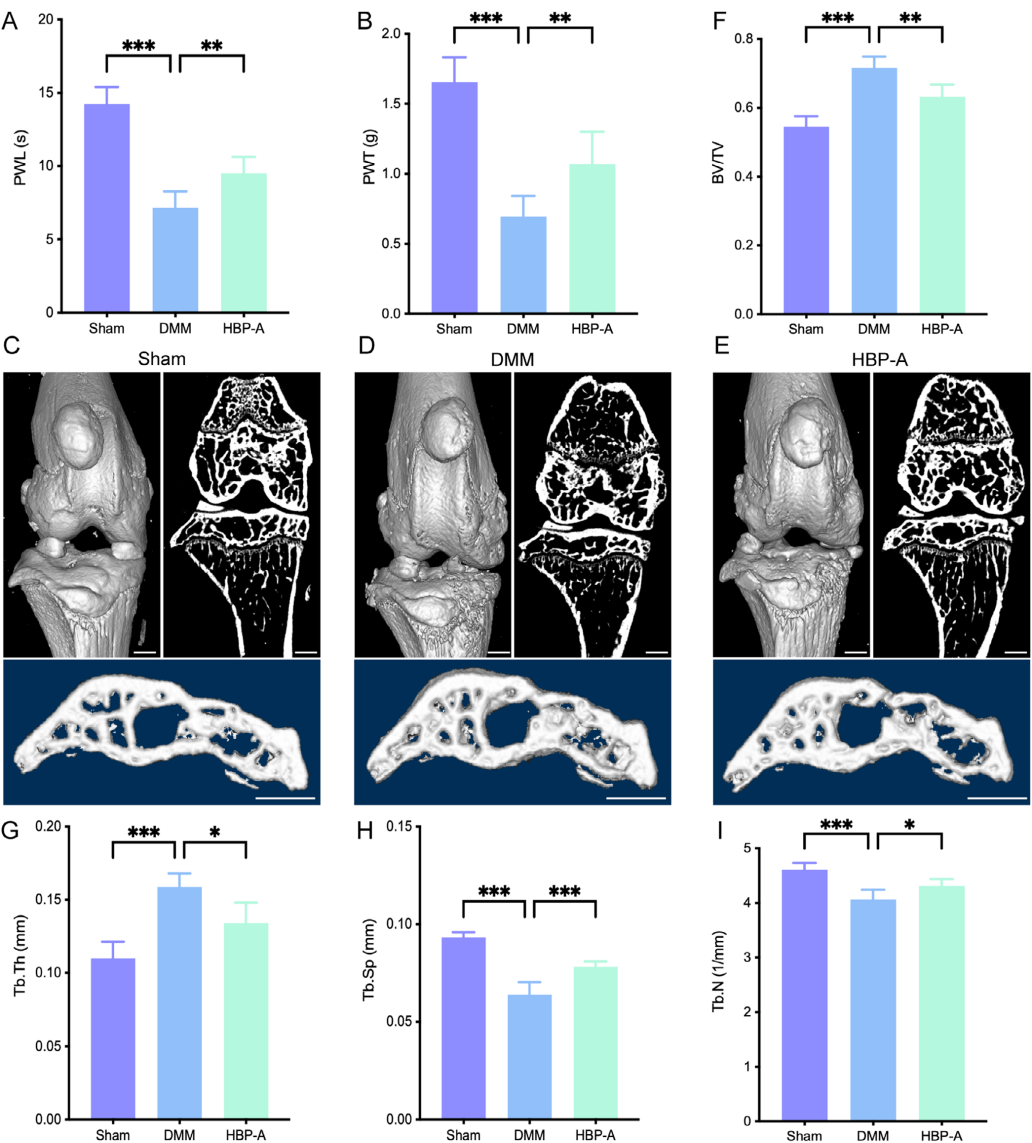
HBP‐A alleviates the pain and OA development in the DMM‐induced mice model. (A and B) Pain‐related behaviours and quantitative analysis of PWL and PWT. (C–E) Representative 2D and 3D reconstruction images of the knee joint and a 3D microscopic view of the medial tibial subchondral bone. Scale bar: 1 mm. (F–I) Quantitative analysis of the BV/TV, Tb. Th, Tb. Sp, and Tb.N. Data were presented as means ± S.D. (*n* = 6 in each group). *p < 0.05, **p < 0.01, ***p < 0.001; BV/TV, bone volume/tissue volume; DMM, destabilisation of the medial meniscus; HBP‐A, Hyriopsis Bioactive Polysaccharide‐Anodonta; PWL, paw withdrawal latency; PWT, paw withdrawal threshold; Tb.N, trabecular number; Tb.Sp, trabecular separation; Tb.Th, trabecular thickness.

Pain is the most prominent and common symptom of OA, progressively worsening as the disease advances. As shown in Figure 1A,B, 4 weeks post‐surgery, the DMM group exhibited significantly reduced PWL and PWT compared to the Sham group (*p* < 0.001, respectively). However, HBP‐A treatment markedly increased both PWL and PWT (*p* < 0.01, respectively), suggesting that HBP‐A effectively alleviates pain in KOA mice.

Subchondral bone remodelling is a hallmark of KOA progression. To evaluate the effect of HBP‐A on subchondral bone microarchitecture, micro‐CT analysis was performed. Three‐dimensional (3D) images revealed notable subchondral bone changes in the DMM group, including increased osteophyte formation and subchondral bone sclerosis, compared to the Sham group (Figure 1C–E). Quantitative analysis demonstrated significantly elevated BV/TV and Tb.Th (*p* < 0.001, respectively), while Tb.Sp and Tb.N were markedly reduced (*p* < 0.001, respectively). After 4 weeks of HBP‐A intervention, these pathological changes were significantly ameliorated. Specifically, BV/TV and Tb.Th were reduced in the HBP‐A group (*p* < 0.01 and *p* < 0.05, respectively), while Tb.Sp and Tb.N increased significantly (*p* < 0.001 and *p* < 0.05, respectively) compared to the DMM group (Figure 1F–I). These findings highlight the potential of HBP‐A to mitigate subchondral bone abnormalities associated with KOA.

### 3.2 HBP‐A Alleviated Articular Cartilage Degeneration, Extracellular Matrix (ECM) Degradation, and Chondrocyte Apoptosis

**FIGURE 2:**
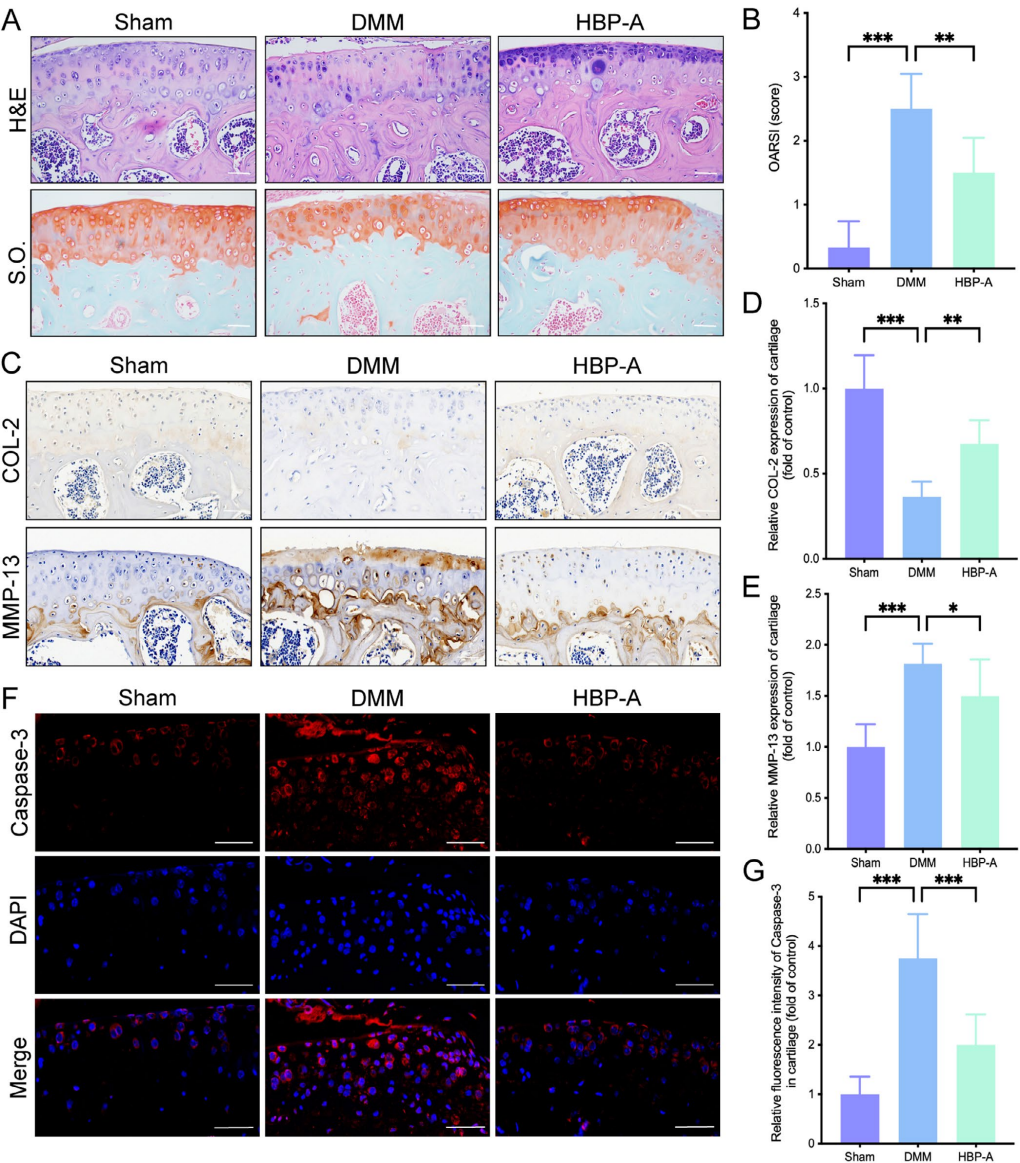
HBP‐A alleviated articular cartilage degeneration, extracellular matrix degradation, and apoptosis in the DMM‐induced mice model. (A) The representative images of H&E and S.O. staining. (B) OARSI scores for the articular cartilage degeneration assessment at 4 weeks. (C–E) Immunohistochemical images and quantificational evaluation of COL‐2 and MMP‐13 expression in articular cartilage at 4 weeks post‐operation. (F and G) Immunofluorescence images and quantificational evaluation of caspase‐3 expression in articular cartilage at 4 weeks post‐operation. Scale bar = 50 μm. Data were presented as means ± S.D. (*n* ≥ 5 in each group). Scale bar = 50 μm. *p < 0.05, **p < 0.01, ***p < 0.001; COL‐2, collagen type II; DMM, destabilisation of the medial meniscus; HBP‐A, Hyriopsis Bioactive Polysaccharide‐Anodonta; MMP‐3, matrix metalloproteinase‐3.

To assess the effect of HBP‐A on articular cartilage homeostasis, histological and IHC analyses were conducted. H&E and S.O. staining were performed to evaluate cartilage morphology and proteoglycan content. As expected, the DMM group exhibited a significantly elevated OARSI score (*p* < 0.001), characterised by substantial proteoglycan loss and surface erosion. In contrast, HBP‐A treatment mitigated these changes, improving cartilage thickness and restoring surface smoothness, thus demonstrating its protective effects on cartilage integrity (*p* < 0.01, Figure 2A,B).

To further investigate whether HBP‐A alleviates ECM degradation in chondrocytes, we evaluated key markers associated with ECM remodelling. IHC results showed a significant decrease in COL‐2 and a marked increase in MMP13 expression in the DMM group (*p* < 0.001, respectively), indicating accelerated ECM degradation. However, HBP‐A treatment reversed these changes, increasing COL‐2 levels and reducing MMP13 expression (*p* < 0.01, *p* < 0.05, respectively; Figure 2C–E).

Additionally, chondrocyte apoptosis is a critical contributor to KOA pathogenesis. To examine the anti‐apoptotic effects of HBP‐A, IF staining was performed to detect caspase‐3 expression in articular cartilage. Compared to the Sham group, the DMM group exhibited significantly elevated caspase‐3 levels (*p* < 0.001, Figure 2F,G). Notably, HBP‐A treatment significantly reduced caspase‐3 expression (*p* < 0.001), demonstrating its anti‐apoptotic effects. Collectively, these findings indicate that HBP‐A exerts chondroprotective effects by attenuating ECM degradation, reducing chondrocyte apoptosis, and preserving cartilage homeostasis in vivo.

### 3.3 HBP‐A Improved Wet Weight, Morphology, Apoptosis, and Ultrastructure of the Quadriceps in KOA

**FIGURE 3:**
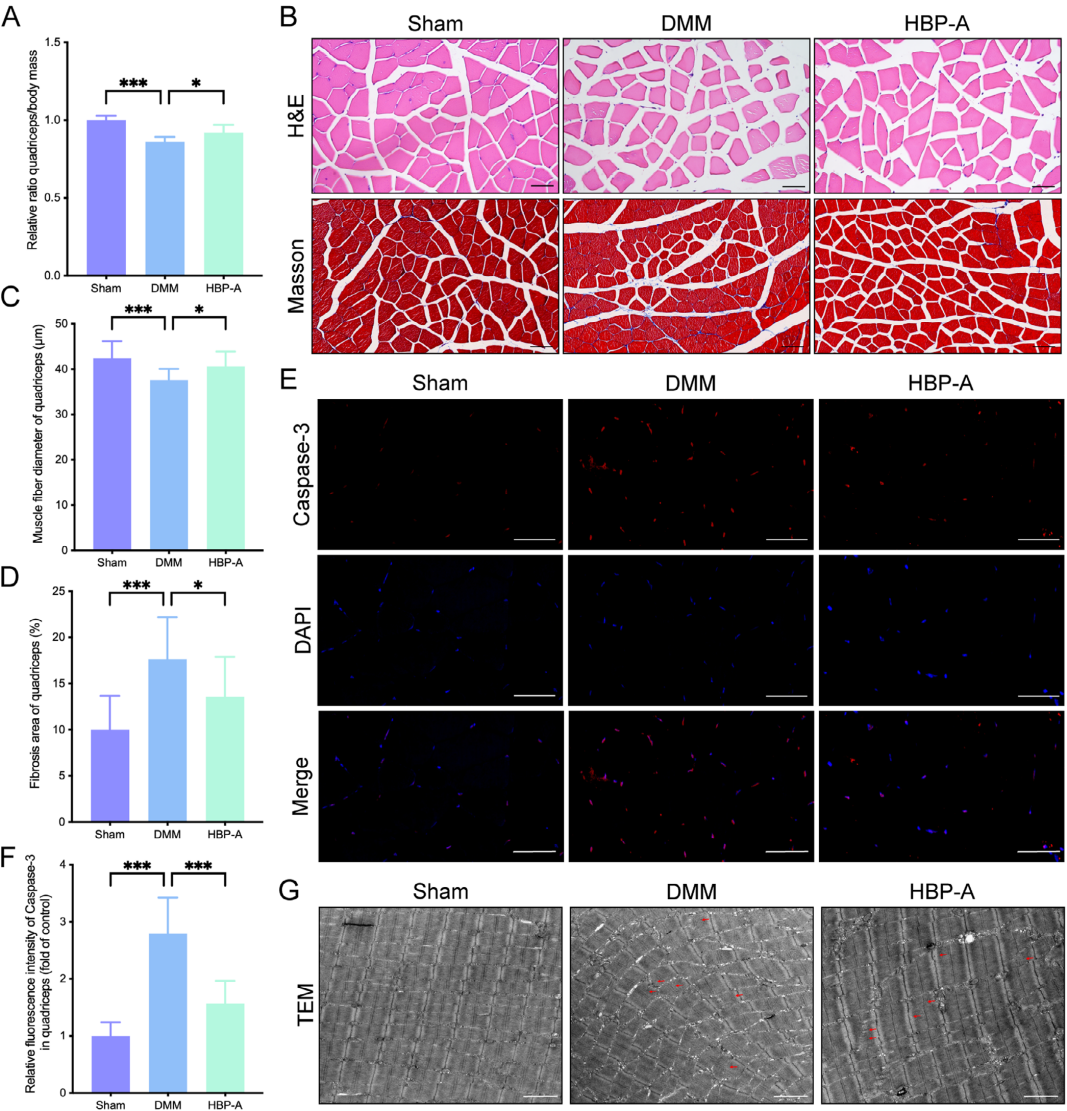
HBP‐A improved wet weight, morphology, apoptosis, and ultrastructure of quadriceps in the DMM‐induced mice model. (A) Quantitative data of quadriceps wet weight at 4 weeks after DMM. (B) The representative images of H&E and Masson staining. Scale bar = 50 μm. (C and D) Quantitative data of muscle fibre diameter and collagen fibre area of the quadriceps. (E and F) Immunofluorescence images and quantificational evaluation of caspase‐3 expression in quadriceps at 4 weeks after DMM. Data were presented as means ± S.D. (*n* ≥ 5 in each group). Scale bar = 50 μm. *p < 0.05, **p < 0.01, ***p < 0.001; (G) Representative images of the muscle fibre ultrastructure of the quadriceps. Red arrows indicated muscle fibres with ultrastructural damage. Scale bar = 2 μm. DMM, destabilisation of the medial meniscus; HBP‐A, Hyriopsis Bioactive Polysaccharide‐Anodonta.

To evaluate the effects of HBP‐A on the quadriceps, we analysed wet weight, morphology, apoptosis, and ultrastructure. H&E and Masson staining revealed significant quadriceps atrophy in the DMM group, as evidenced by reduced wet weight and muscle fibre diameter, accompanied by increased collagen fibre area (*p* < 0.001, respectively). HBP‐A treatment significantly restored quadriceps wet weight and muscle fibre diameter (*p* < 0.05; Figure 3A–D), reducing collagen fibre accumulation. IF analysis demonstrated elevated caspase‐3 expression in the DMM group (*p* < 0.001), indicating increased apoptosis. HBP‐A treatment markedly reduced Caspase‐3 levels (*p* < 0.001; Figure 3E,F), suggesting its role in suppressing apoptotic processes in the quadriceps. Besides, TEM analysis was employed to evaluate the quadriceps ultrastructure. The DMM group exhibited significant abnormalities, including disrupted Z‐lines and sarcomere disorganisation, indicative of impaired muscle integrity. HBP‐A treatment improved ultrastructural features, restoring Z‐line alignment and sarcomere organisation (Figure 3G).

In summary, HBP‐A effectively alleviated quadriceps atrophy, reduced apoptosis, and restored ultrastructural integrity in the DMM‐induced KOA model.

### 3.4 HBP‐A Regulated Bone Factors Driven by the Quadriceps in KOA

**FIGURE 4:**
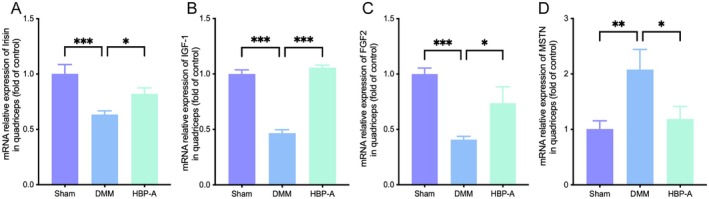
HBP‐A regulated bone factors driven by the quadriceps in DMM mice. (A–D) The gene expression of quadriceps treated with HBP‐A in the DMM‐induced mice model. (A) Irisin; (B) IGF‐1; (C) FGF‐2; (D) MSTN; Data were presented as means ± S.D. (*n* ≥ 3 in each group); *p < 0.05, **p < 0.01, ***p < 0.001; DMM, destabilisation of the medial meniscus; FGF‐2, fibroblast growth factor 2; HBP‐A, Hyriopsis Bioactive Polysaccharide‐Anodonta; IGF‐1, insulin‐like growth factor 1; MSTN, myostatin.

Muscle‐bone crosstalk is critical in maintaining joint homeostasis, with its dysregulation contributing to KOA progression. RT‐qPCR analysis revealed that the expression of beneficial bone factors, including Irisin, IGF‐1, and FGF‐2, was significantly downregulated in the DMM group compared to the Sham group (*p* < 0.001 for all, Figure 4A–C). Conversely, MSTN, a negative regulator of bone metabolism, was markedly upregulated in the DMM group (*p* < 0.01, Figure 4D). In contrast, the HBP‐A group exhibited opposite trends: HBP‐A treatment significantly upregulated Irisin, IGF‐1, and FGF‐2 expression (*p* < 0.05, *p* < 0.001, *p* < 0.05, respectively) while reducing MSTN expression compared to the DMM group (*p* < 0.05).

### 3.5 HBP‐A Inhibited the MLK3/P38 Signalling Pathway in Articular Cartilage and Quadriceps of KOA Mice

**FIGURE 5:**
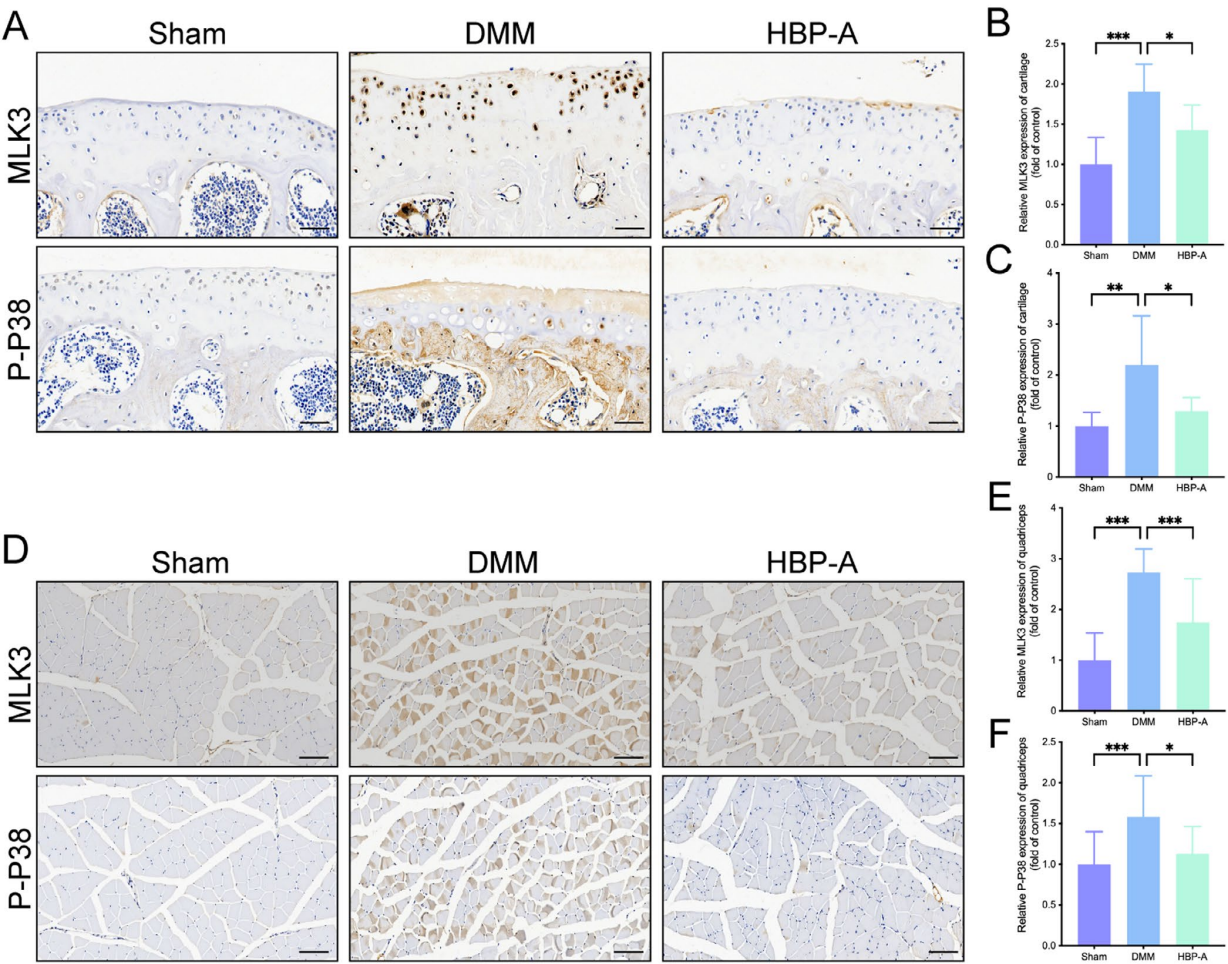
HBP‐A inhibited the MLK3/P38 MAPK signalling pathway in articular cartilage and quadriceps in the DMM‐induced mice model. (A, D) Representative immunohistochemical images of MLK3 and P‐P38 in the articular cartilage and quadriceps at 4 weeks, respectively; (B and C) Quantitative analyses of the expression of MLK3 and P‐P38 in the articular cartilage. (E and F) Quantitative analyses of the expression of MLK3 and P‐P38 in the quadriceps. Data were presented as means ± S.D. (*n* ≥ 5 in each group); scale bar = 50 μm. *p < 0.05, **p < 0.01, ***p < 0.001; DMM, destabilisation of the medial meniscus; HBP‐A, Hyriopsis Bioactive Polysaccharide‐Anodonta; MLK3, mixed‐lineage kinase 3; P‐P38, phosphorylated‐p38.

IHC staining was used to evaluate the expression of MLK3 and P‐P38 MAPK in the articular cartilage and the quadriceps. The DMM group showed significantly elevated MLK3 and P‐P38 MAPK expression in both tissues compared to the Sham group (*p* < 0.05 for all). However, HBP‐A treatment effectively reduced MLK3 and P‐P38 MAPK expression in both articular cartilage and quadriceps compared to the DMM group (*p* < 0.05 for all; Figure 5), reversing the pathological upregulation.

### 3.6 HBP‐A Enhanced the HDAC4 Expression in Articular Cartilage and Quadriceps in the DMM‐Induced Mice Model

**FIGURE 6:**
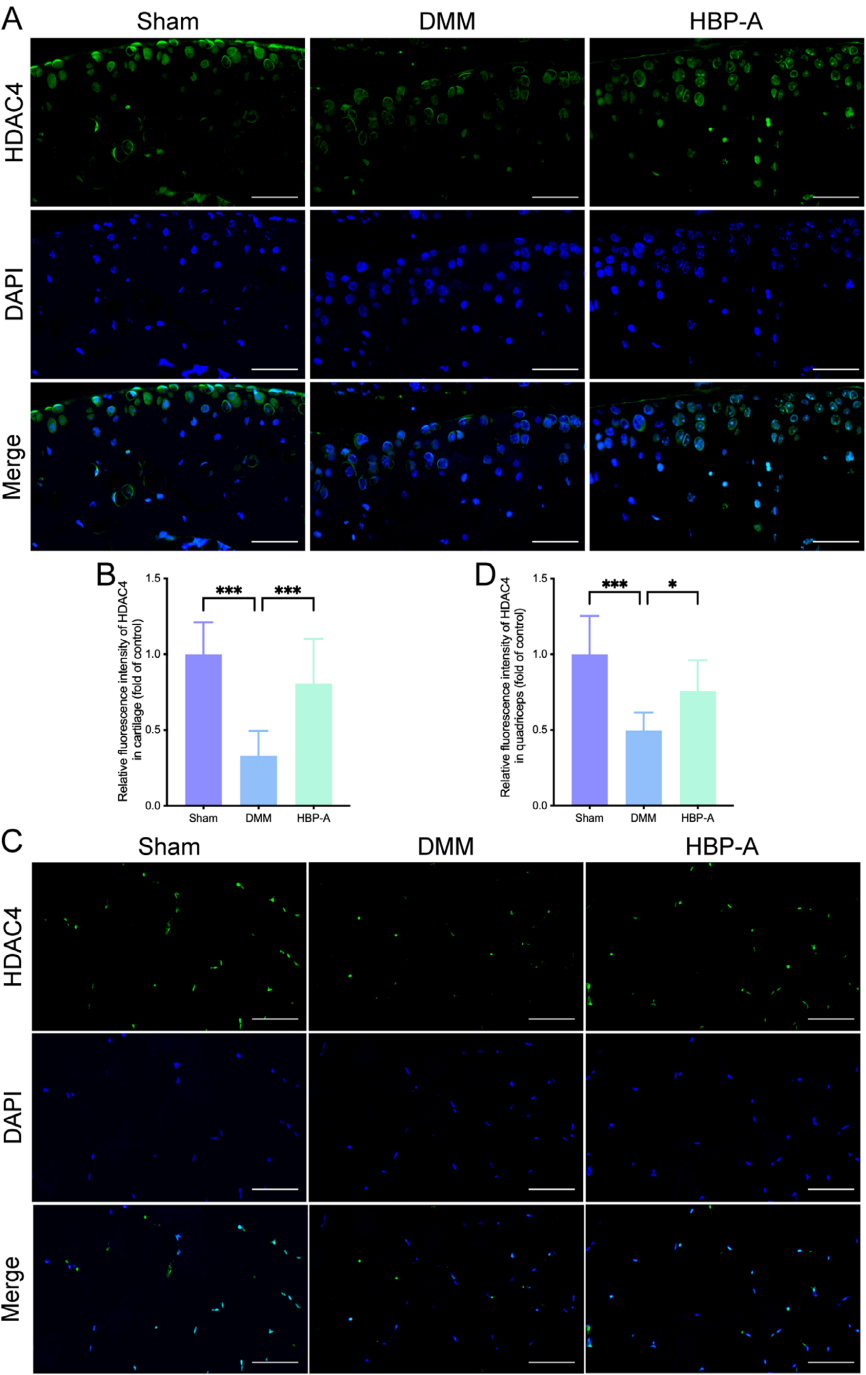
HBP‐A enhanced the HDAC4 expression in articular cartilage and quadriceps in the DMM‐induced mice model. (A, B) Immunofluorescence images and quantification evaluation of HDAC4 expression in the articular cartilage at 4 weeks after DMM. (C, D) Immunofluorescence images and quantification evaluation of HDAC4 expression in the quadriceps at 4 weeks after DMM. Data were presented as means ± S.D. (*n* ≥ 5 in each group); scale bar = 50 μm. *p < 0.05, **p < 0.01, ***p < 0.001; DMM, destabilisation of the medial meniscus; HBP‐A, Hyriopsis Bioactive Polysaccharide‐Anodonta; HDAC4, histone deacetylase 4.

IF staining revealed significantly reduced HDAC4 expression in both the articular cartilage and quadriceps of the DMM group compared to the Sham group (*p* < 0.001 for both; Figure 6). Notably, HBP‐A treatment maintained a higher HDAC4 level in both tissues (*p* < 0.001 and *p* < 0.05, respectively).

## 4 Discussion

KOA is a progressive and degenerative disease with limited treatment options, particularly in its advanced stages, where joint replacement surgery is often the most effective intervention [41]. However, such procedures are generally reserved for end‐stage KOA due to associated risks and complications, making them unsuitable for early intervention [42]. For mild to moderate KOA, the primary therapeutic goals are to alleviate symptoms, preserve joint function, and delay or prevent disease progression [43]. Recently, TCM has garnered significant attention for its multi‐targeted therapeutic effects [31]. HBP‐A, a proprietary prescription extract developed at our centre using mussel meat, has demonstrated clinical efficacy and promising therapeutic potential. Previous studies [9, 44] have shown that HBP‐A can inhibit the activation of the P38 MAPK pathway and upregulate the expression of HDAC4 in meniscal fibrochondrocytes under abnormal mechanical stress. However, its effects on articular cartilage and quadriceps muscle—both essential for maintaining knee joint stability—remain poorly understood in vivo. In this study, we employed a DMM‐induced KOA model to investigate the mechanisms underlying the therapeutic effects on articular cartilage and quadriceps muscle under mechanical instability. Using a variety of analytical methods, our research provides comprehensive insights into how HBP‐A mitigates KOA progression.

KOA is characterised by key pathological features, including joint matrix degradation, osteophyte formation, and cartilage degeneration [41]. In this study, HBP‐A significantly alleviated pain and abnormal subchondral bone remodelling in the DMM‐induced KOA model. Subchondral bone plays a critical role in mechanical force transmission across the joint [45]. Abnormal mechanical stress due to subchondral bone remodelling contributes to joint inflammation and pain, which are hallmarks of KOA [46]. By mitigating aberrant subchondral bone metabolism, HBP‐A likely restored joint mechanical homeostasis, alleviated pain, and improved overall knee joint functionality.

The ECM is critical for maintaining the structural integrity and biological functionality of articular cartilage [47]. Its equilibrium is regulated by the anabolic and catabolic activities of chondrocytes. OA disrupts this balance, resulting in increased ECM degradation and reduced synthesis of its components [48]. COL‐2, a fundamental structural protein in the cartilage ECM, is a key indicator of ECM metabolism. In contrast, MMP‐13, a potent catabolic enzyme responsible for COL‐2 degradation, is typically expressed at low levels in healthy cartilage. Immunohistochemical analysis in this study demonstrated that HBP‐A effectively suppressed MMP‐13 expression while restoring COL‐2 levels in the KOA model. These results indicate that HBP‐A exerts therapeutic effects by delaying ECM degradation and promoting cartilage homeostasis. Furthermore, HBP‐A's ability to modulate subchondral bone metabolism and ECM dynamics collectively highlights its potential to inhibit the pathological progression of KOA.

The soft tissues surrounding the joint, particularly the quadriceps, play a pivotal role in maintaining mechanical stability [49]. This study comprehensively evaluated the quadriceps to assess the impact of the HBP‐A intervention. First, the quadriceps' stability is largely determined by the volume and structure of its muscle fibres, with increased muscle fibre wet weight and diameter reflecting enhanced mechanical support [50]. In this study, HBP‐A mitigated degenerative changes in the quadriceps induced by DMM, as evidenced by improvements in muscle fibre diameter, wet weight, and ultrastructural integrity. Specifically, HBP‐A preserved the organisation of Z‐lines and sarcomeres, which are essential for muscle force generation. Second, the pathological transformation of muscle fibres into collagen fibres significantly impairs quadriceps function. Excessive collagen fibre accumulation reduces muscle strength and compromises joint stability [51]. The findings demonstrated that HBP‐A effectively prevents quadriceps fibrosis, thereby preserving its functional capacity. These observations align with previous studies [52, 53] emphasising the importance of limiting muscle fibrosis to maintain joint stability. Finally, emerging evidence suggests that muscle acts as an endocrine organ, secreting cytokines that regulate bone growth and metabolism, which are crucial in KOA progression [54]. Notably, HBP‐A significantly increased the expression of Irisin, FGF‐2, and IGF‐1 while reducing MSTN. These alterations enhance subchondral bone metabolism and may contribute to delaying KOA progression. Collectively, these findings underscore the multifaceted benefits of HBP‐A in preserving the structural and functional integrity of the quadriceps, mitigating cartilage degeneration, and delaying KOA progression.

Apoptosis, a genetically regulated process of programmed cell death, is essential for maintaining cellular homeostasis by complementing mitosis under normal physiological conditions. Together, these processes ensure tissue integrity and cell renewal. However, under pathological or ageing conditions, elevated rates of apoptosis disrupt this delicate balance [55]. In KOA, excessive apoptosis accelerates the degeneration of articular cartilage and skeletal muscle, exacerbating disease progression [56]. Our findings revealed that HBP‐A treatment significantly reduced the expression of caspase‐3, recognised as a central executioner of apoptosis and a critical biomarker for programmed cell death, in the articular cartilage and quadriceps of DMM‐induced osteoarthritis mice.

These results demonstrate that HBP‐A suppresses apoptosis, thereby preserving the structural integrity and functional roles of these critical joint tissues in maintaining stability and mobility. This aligns with previous studies emphasising the therapeutic potential of anti‐apoptotic strategies in managing degenerative joint diseases [57]. The observed anti‐apoptotic effects of HBP‐A may be attributed to its modulation of signalling pathways, particularly the MLK3/P38 MAPK pathway in this study.

The activation of signalling pathways and the regulation of related genes, proteins, and growth factors are crucial in the pathological and reparative processes of cartilage and skeletal muscle [58, 59, 60]. Among these, the P38 MAPK signalling pathway is pivotal in intracellular signal transduction, mediating bone and muscle growth, gene expression, and cellular responses to environmental stress [58]. In skeletal muscle, activation of the P38 MAPK pathway leads to phosphorylation of target residues, triggering muscle satellite cell activation [61]. These cells re‐enter the cell cycle, contributing to muscle fibre formation and repair. However, under external stressors such as inflammatory factors, cellular injury, or hypoxia, overactivation of the P38 MAPK pathway induces elevated levels of cyclin‐dependent kinase inhibitors (e.g., p21, p16), which impair satellite cell proliferation and hinder muscle regeneration [62].

The MAPK pathway operates through a cascade of activations involving MAPK kinase kinases (MAP3Ks), MAPK kinases (MAP2Ks), and MAPKs, with dual‐site phosphorylation critical for nuclear translocation and transcription factor activation [63]. MLK3, a serine/threonine protein kinase, functions as an upstream regulator of the p38 MAPK pathway and is widely expressed in tissues such as skeletal muscle, lung, liver, and heart, where it mediates diverse biological functions [24]. In this study, we found significantly elevated expression levels of MLK3 and P‐P38 MAPK in the cartilage and quadriceps of the DMM group. HBP‐A treatment markedly reduced these protein levels, suggesting its protective effects against cartilage and quadriceps degeneration. These findings highlight the potential of targeting the MLK3/P38 MAPK pathway as a therapeutic strategy for KOA.

HDAC4 serves as a crucial epigenetic regulator of gene expression and cellular physiology, primarily mediating chromatin remodelling and transcriptional silencing to maintain tissue homeostasis [64]. Beyond its fundamental biological functions, HDAC4 demonstrates tissue‐specific essentiality in preserving the structural and functional integrity of articular cartilage and skeletal muscle systems [65, 66]. In cartilage, HDAC4 regulates chondrocyte differentiation and extracellular matrix turnover, contributing to cartilage homeostasis [44, 66]. Concurrently, in skeletal muscle, HDAC4 orchestrates adaptive responses to mechanical stress by coordinating myofibre repair mechanisms and regenerative pathways [65].

During KOA progression, HDAC4 expression is significantly reduced in both articular cartilage and quadriceps muscle, which disrupts normal cellular processes in articular cartilage and quadriceps. This downregulation exacerbates cartilage matrix breakdown and muscle degeneration, further accelerating disease progression. Our study demonstrated that HBP‐A treatment effectively counteracts KOA‐induced HDAC4 depletion in the cartilage and quadriceps of DMM models. These observations substantiate HDAC4 preservation as a central mechanism through which HBP‐A modulates multiple pathological axes in KOA progression.

This study systematically elucidates the chondroprotective and myoprotective efficacy of HBP‐A in a biomechanically induced KOA model, demonstrating its capacity to modulate the MLK3/P38 MAPK/HDAC4 signalling axis. While our findings advance understanding of HBP‐A's therapeutic potential, three critical limitations warrant consideration. First, the current experimental framework did not incorporate functional assessments through behavioural phenotyping (e.g., dynamic gait analysis) or advanced imaging modalities (e.g., μMRI, ultrasound elastography), which could objectively quantify improvements in joint mechanics and muscle contractility. Second, while we established HBP‐A's regulatory effects on the MLK3/P38/HDAC4 cascade, the absence of gene expression profiling (e.g., RT‐qPCR/RNA‐seq for MLK3, HDAC4 isoforms) leaves unresolved whether HBP‐A directly modulates transcriptional regulation of these pathway components or acts through post‐translational mechanisms. Third, the therapeutic scope of HBP‐A may extend beyond this identified axis, potentially involving cross‐talk with complementary pathways such as Wnt/β‐catenin or TGF‐β/Smad signalling, which were not investigated in this study.

Future investigations should prioritise multi‐modal functional assessments to correlate molecular changes with biomechanical outcomes, coupled with mechanistic studies employing techniques like chromatin immunoprecipitation (ChIP‐seq) and RNA interference to dissect HBP‐A's precise molecular targets. Additionally, systems‐level approaches integrating transcriptomic and proteomic analyses could unveil novel signalling networks modulated by HBP‐A. Addressing these knowledge gaps through rigorous multi‐omics methodologies and functional validations will be essential for translating these preclinical findings into targeted therapeutic strategies for KOA management.

## 5 Conclusion

This study demonstrates that HBP‐A alleviates knee osteoarthritis progression by simultaneously preserving articular cartilage integrity and mitigating quadriceps atrophy through inhibition of the MLK3/P38 MAPK/HDAC4 axis.

## Author Contributions

**Zhengming Wang:** writing – original draft (equal). **Junyan Huang:** writing – original draft (equal). **Yuanyuan Feng:** writing – original draft (equal). **Zhengyan Li:** methodology (equal). **Haiya Ge:** methodology (equal). **Rui Wang:** formal analysis (equal). **Yong Gu:** visualization (equal). **Yizhe Xiong:** visualization (equal). **Bo Chen:** methodology (equal). **Mingcai Zhang:** formal analysis (equal). **Xiang Wang:** writing – review and editing (equal). **Ying Shi:** writing – review and editing (equal). **Zhibi Shen:** methodology (equal). **Hongsheng Zhan:** supervision (equal). **Guoqing Du:** funding acquisition (equal), supervision (equal).

## Ethics Statement

All the animal operating procedures were approved by the Experimental Animal Ethics Committee of Shanghai University of Traditional Chinese Medicine (PZSHUTCM2306260005).

## Conflicts of Interest

The authors declare no conflicts of interest.

## Supporting information


Table S1.

## Data Availability

The original contributions presented in the study are included in the article; further enquiries can be directed to the corresponding author.
