# Supplementary material for: HBP‐A Attenuates Knee Osteoarthritis Progression via MLK3/P38/HDAC4 Axis‐Mediated Dual Protection of Articular Cartilage and Quadriceps
Source: J Cell Mol Med. 2025 May 3;29(9):e70577. doi: 10.1111/jcmm.70577 (PMC12049151; doi:10.1111/jcmm.70577)
Supplement: Supplementary file 1 — Table S1. [file JCMM-29-e70577-s001.docx]

Supplementary Table 1 Primer name and sequences for PCR analysis.

| Primer name | Sequences |
| --- | --- |
| *β*-actin forward | 5’-GGAGATTACTGCCCTGGCTCCTA-3’ |
| *β*-actin reverse | 5’-GACTCATCGTACTCCTGCTTGCTG-3’ |
| Irisin forward | 5’-AGGATGAAGTGGTCATTGGCTTTG-3’ |
| Irisin reverse | 5’-CCTTGTTGTTATTGGGCTCGTTG-3’ |
| MSTN forward | 5’-AGTGGATCTAAATGAGGGCAGT-3’ |
| MSTN reverse | 5’-GGAGTACCTCGTGTTTTGTCTC-3’ |
| IGF-1 forward | 5’-ACAAGCCCACAGGCTATGGCTC-3’ |
| IGF-1 reverse | 5’-AGTCTCCTCAGATCACAGCTCCG-3’ |
| FGF2 forward | 5’-CACCAGGCCACTTCAAGGA-3’ |
| FGF2 reverse | 5’-GATGGATGCGCAGGAAGAA-3’ |
